# Inequalities in children’s mental health prescribing and referrals for specialist mental health services.

**DOI:** 10.23889/ijpds.v7i3.1980

**Published:** 2022-08-25

**Authors:** William Ball, Helen Rowlands, Corri Black, Shantini Paranjothy, Adelene Rasalam, David Ritchie, Magdalena Rzewuska, Elaine Thompson, Katie Wilde, Jessica Butler

**Affiliations:** 1 University of Aberdeen; 2 NHS Grampian

## Objectives

1 in 8 young people in the United Kingdom are estimated to have a diagnosable mental health condition. Prevalence is increasing over time, many are untreated, and need is not evenly distributed across the population. We aimed to investigate trends in children’s mental health prescribing and referrals to specialist outpatient services.

## Approach

We linked individual-level healthcare administrative records on community prescribing and referrals to outpatient Child and Adolescent Mental Health Services (CAMHS). The study cohort included all children aged 2 through 17 in the NHS Grampian Health Board region from 2015 to 2021 (average annual population circa 100,000) with a mental health prescription or CAMHS referral.

We measured prevalence of mental health prescribing and referrals to CAMHS over time. We investigated demographic and socioeconomic differences, including comparison of rates by age, sex, and residential area deprivation. We also investigated socioeconomic and demographic differences in referral acceptance and rejection.

## Results

Prescriptions for mental health drugs have risen 40%: from 5,000 per month in 2015 to 7,000 in 2021. 75% of prescriptions to primary schoolers are to boys, mostly for attention deficit hyperactivity disorder medications. Prescriptions to girls rise during secondary school, mostly for anti-depressants. Prescribing rates are 2.6-fold higher in the most versus least deprived areas.

Referrals to CAMHS have risen 20% over the study period, and the proportion of referrals rejected has increased from 18% to 31% – leaving the number of children accepted to specialist care stable. Boys are referred twice as often at younger ages, while girls’ referrals spike during puberty. Since 2015, boys have been referred less and rejected more, with girls now making up 61% of those treated. Referral rates are two-fold higher in the most versus least deprived areas.

## Conclusion

Both mental health prescribing and referrals to CAMHS have increased in this population, but the CAMHS service size remained fixed. Presentation and treatment patterns vary dramatically by age and sex, and socioeconomic inequalities are clear and persistent.

